# Logotherapy techniques to unlock resilience among Ukrainian refugees: a pre-post quasi-experimental design

**DOI:** 10.3389/fpsyt.2026.1829381

**Published:** 2026-06-12

**Authors:** Andriy Haydabrus, Lydia Giménez-Llort

**Affiliations:** 1 Department of Psychiatry and Forensic Medicine, School of Medicine, Universitat Autònoma de Barcelona, Barcelona, Spain; 2 NGO “Complex Psychology Help”, Kharkiv, Ukraine; 3 Institut de Neurociències, Universitat Autònoma de Barcelona, Barcelona, Spain

**Keywords:** anxiety disorders, depressive disorder, logotherapy, migration, psychotherapy, refugees, Ukraine, meaning-centered psychological support

## Abstract

The ongoing Russian-Ukrainian war has triggered a significant refugee crisis, resulting in widespread trauma, displacement, and mental health challenges among affected populations. This study aimed to explore the potential usefulness of logotherapy, a meaning-centered therapy, in addressing the mental health needs of Ukrainian refugees. The research was conducted in a naturalistic scenario of a group of 20 Ukrainian refugees residing in Europe who received a tailored logotherapy or meaning-centered psychological support, compared with controls who did not. Meaning-oriented techniques, namely, Socratic Dialogue, Modification of Attitude, Paradoxical Intention, and Dereflection were used during the intervention. Participants were assessed in a pre-post quasi-experimental design using validated self-report measures for anxiety (Generalized Anxiety Disorder-7), depression (Beck Depression Inventory), and general health (General Health Questionnaire-12). The improvements were clearly observed as reductions in their self-reported anxiety and depressive symptoms after the meaning-centeres psychological support. Overall, these preliminary findings indicate that logotherapy may be a promising and feasible approach to psychological support for refugee populations. However, given its pilot nature and quasi-experimental design, causal conclusions cannot be drawn, and further research using larger, randomized, and methodologically rigorous designs is warranted to examine long-term effects and broader applicability.

## 1 Introduction

The Lancet Psychiatry Commission on mental health in Ukraine (2024) warned about the combatant population at high risk of psychological trauma and brain injuries. Our previous work on lessons from the impact of war on combatants’ mental health during the last decade (1) was one of the four cited reports in this respect. Nevertheless, as anticipated by the UCL-Lancet Commission on Migration and Health report on the secondary impact of wars (2), meeting the health challenges of displaced populations from Ukraine (3), with special attention to addressing war trauma before being too late, was a huge challenge too (4).

The ongoing war in Ukraine has triggered the most significant refugee crisis in Europe since World War 2, with millions of people expected to flee the country: 8, 163, 268 refugees from Ukraine were recorded across Europe from February 24 to October 15, 2024. Among them, 6, 191, 800 refugees from Ukraine registered for Temporary Protection or similar national protection schemes in Europe (5). They are vulnerable to distress and mental health problems. Stigma, difficulties in adapting, cultural barriers, and reduced access to mental health support can seriously hamper their ability to build resilience and recover. The impact of war, trauma, and devastation is dire, necessitating urgent action to address the mid-term and long-term needs of the affected populations (6). This includes a swift response from all sectors, especially the health sector, to address the consequences of this crisis.

At the beginning of the war, postulates were formulated to prevent a humanitarian crisis due to the millions of refugees (3). They consisted of an immediate cessation of military aggression by the Russian government. Initially, these postulates included allowing the evacuation of patients from hospitals, refraining from attacking healthcare institutions, the Ukrainian government not hindering the migration of disabled and medically vulnerable individuals, ensuring continuity of medical care for people with chronic illnesses during evacuation, entitling medical workers to work in the countries where they migrated, and providing necessary assistance in supplying Ukraine with essential drugs and equipment (3). Today, we observe that not all conditions have been met, and the fighting continues.

A systematic review and meta-analysis of prevalence studies (all based on clinical interviews and validated diagnostic systems) reported that, overall, 31.5% of refugees had post-traumatic stress disorder (PTSD), 31.5% had depressive disorders, 11.1% had anxiety disorders, and 1.5% had psychoses (7). The rates of PTSD and depression in the group of refugees are dramatically higher than those in the general population. The nature of post-traumatic stress disorder (PTSD) among refugee populations should be investigated using cross-cultural, longitudinal, and experimental research paradigms to better understand the underlying mechanisms. Currently, there is a lack of comprehensive data regarding PTSD in refugees, highlighting the need for focused research efforts to enhance treatment effectiveness and reduce barriers to accessing modern mental healthcare (8). Those not displaying signs and symptoms now will carry a greater allostatic load and are likely to suffer poor physical and mental health in the long term. A significant proportion of refugees will have alcohol and substance use disorders, which may be precipitated or aggravated by trauma (7). The diagnosis of the mental health of refugees has yielded such results that we cannot ignore during the forecast of the development of events with refugees from Ukraine. The Ukrainian refugee crisis highlights the many issues associated with trauma, distress, and mental health (9).

Despite the UCL-Lancet Commission on Migration and Health report offering evidence-based strategies to meet the health needs of forcibly displaced persons (2), countries receiving large numbers of Ukrainian refugees have faced challenges, as many refugees are unfamiliar with the structure of psychological support and encounter both linguistic and cultural barriers (9). While there is a critical need for trauma-related psychological care, psychiatrists and primary care providers have encountered fewer difficulties when treating depression and anxiety through medication (4).

The foundations and applications of logotherapy to improve the mental health of immigrant populations in the third millennium (10) suggest that logotherapy is especially well-suited for crisis situations. This is because it emphasizes meaning-making in the face of suffering—an approach that resonates with the existential challenges forcibly displaced individuals face. Refugees frequently endure the traumatic loss of home, social ties, and personal security, resulting in deep disruptions to identity and purpose. By guiding individuals to discover enduring values and personal meaning amid adversity, logotherapy addresses critical psychosocial issues such as hopelessness, anxiety, and diminished agency. Developed by Viktor Frankl in response to extreme suffering, the theory underscores that even in uncontrollable circumstances, people retain the freedom to choose their attitudes and find meaning. This empowering perspective can foster resilience and restore a sense of coherence and purpose—key elements of psychological recovery during humanitarian crises. Its demonstrated effectiveness in helping individuals derive meaning from their experiences also extends to a range of mental health challenges commonly encountered by immigrant populations (10, 11).

Although logotherapy has proven to be an effective approach for reducing levels of depression and anxiety among refugees, even in online settings, there remains a shortage of studies exploring its application to immigrant populations more broadly (12). Future studies should focus more on logotherapy applications and on developing effective therapies for diverse immigrant groups (13). Therefore, the present study aimed to develop a culturally adapted logotherapy protocol for Ukrainian migrants and to assess the feasibility and preliminary outcomes related to anxiety and depressive symptoms among displaced individuals during the war in Ukraine. The first step towards achieving this goal was the implementation of a pilot study with a small group of participants using a pre-post quasi-experimental design. Online and offline groups receiving logotherapeutic intervention were compared with a control group. The aim was to assess the impact of logotherapy on mental health and its potential to address the uncertain current reality in their complex intercultural relationships (13).

## 2 Methods

### 2.1 Study design

This study employed a pre-post quasi-experimental design with two groups from the same general refugee population: a treatment group that received logotherapy and a control group that received no psychological intervention. Due to the quasi-experimental design, group assignment was non-randomized, and no baseline matching for mental health status or demographic characteristics was performed; however, comparative demographic and clinical data were analyzed to identify potential differences.

### 2.2 Design of a tailored logotherapy intervention

#### 2.2.1 Intervention manual availability

Intervention was based on a structured manual derived from prior logotherapy protocols and adapted for this study. The logotherapy intervention, following the standardized manual based on Viktor Frankl’s principles of logotherapy, was adapted from a previous protocol originally designed for displaced populations (14) and specifically tailored to the Ukrainian refugee context.

#### 2.2.2 Group size per session and number of groups delivered

The intervention was delivered in small-group format, with groups intentionally kept small (approximately 6–9 participants per session) to facilitate discussion and reflective exercises, and that multiple groups were delivered to accommodate participant availability and delivery mode.

#### 2.2.3 Delivery mode

The intervention used an in person delivery model for participants who live in Spain and online for participants from other countries. Participants were assigned to the available format based on feasibility rather than random allocation.

#### 2.2.4 Session attendance and homework adherence

Session attendance was monitored via participation records, and retention was high among those who completed the intervention (20/23 completed the post-intervention assessment). While structured quantitative metrics of homework adherence were not prospectively collected in this pilot, between-session reflective exercises and meaning-oriented practices were encouraged and qualitatively monitored during session discussions. This limitation has now been acknowledged.

#### 2.2.5 Facilitator qualifications, supervision, and facilitator consistency

The intervention was delivered by a trained psychologist with experience in clinical work and work with war-affected populations. The same facilitator delivered all intervention sessions to preserve consistency, with professional supervision supporting fidelity to the intervention model.

#### 2.2.6 Facilitator involvement in recruitment and assessment

The facilitator participated in recruitment and intervention delivery and had a role in data collection procedures. Because this may introduce bias, we explicitly acknowledge it as a limitation of the pilot design.

#### 2.2.7 Cultural adaptation development, validation, and community contribution

The intervention was adapted through iterative modifications to examples, themes, and exercises to reflect war-related displacement experiences and Ukrainian cultural meaning systems, drawing on prior logotherapy protocols and the study team’s lived experience. Importantly, Ukrainian refugees themselves contributed to refining the intervention through informal feedback during protocol development and implementation, thereby enabling community-informed adaptation. This cultural adaptation was pragmatic and pilot-oriented rather than formally validated through a separate adaptation framework, which is an area for future work.

#### 2.2.8 Logotherapeutic techniques

To encourage active reflection, challenge assumptions, and foster a shift in participants’ perspectives, four logotherapeutic techniques were employed throughout the sessions, as previously described. Briefly, 1) “Socratic Dialogue” engages individuals in thought-provoking dialogues to facilitate self-reflection and a deeper understanding of their values and meaning (15); 2) “Modification of Attitude” involves altering one’s perspective and attitude towards an unavoidable situation, emphasizing the power of choice in interpreting the situation positively (16); 3) “Paradoxical Intention” encourages individuals to confront their fears or anxieties often diminishes the fear’s hold over them (17); 4) Dereflection, shifts focus from one’s problems by engaging in activities that direct attention away from the problem, aiding in achieving a healthier perspective (17).

### 2.3 Effectiveness of the logotherapy intervention

#### 2.3.1 Participants

Participants were recruited through community centers and refugee support organizations, using snowball and convenience sampling. An explanatory statement was provided via Google Forms, detailing the study’s objectives, voluntary nature, confidentiality protocols, and the right to withdraw at any time. An individual clinical interview was a single session conducted by the primary researcher, a neuropsychologist. After the interview and obtaining informed consent, participants were included in the study if they met the selection criteria as follows:

Inclusion criteria: Ukrainian refugees aged 18–65 years residing in Europe. Individuals experiencing psychological difficulties due to forced displacement from Ukraine because of the war.

Exclusion criteria: Presence of a chronic mental disorder, current use of any psychiatric medications, participation in any other form of psychotherapy at the time of the study. Participants with severe symptoms, suicidality, psychosis, or acute crisis were excluded from the study and referred to appropriate services.

#### 2.3.2 Clinical instruments

Individuals provided information on their socio-demographic profiles and refugee status. To explore the efficacy of the intervention, an initial baseline and a six-week final survey, each consisting of the three questionnaires, were administered to all participants to enable a pre-post analysis. Validated self-reporting questionnaires, namely the Generalized Anxiety Disorder-7 (GAD-7) (18), the Beck Depression Inventory (BDI, 19, 20) to assess the severity of depression symptoms, and the General Health Questionnaire (GHQ-12, 21, 22) were applied. The Ukrainian/Russian versions used have been validated in Ukrainian/Russian-speaking populations and, ideally, among refugees or war-affected populations [The Ukrainian version of GAD-7 and BDI, according to the “Ukrainian Ministry of Health Order No. 2118, 2023” (23); the Russian adaptation of GHQ-12 (24, 25)].

##### 2.3.2.1 Generalized anxiety disorder

The GAD-7 is a 7-item self-report measure that Spitzer and colleagues (18) initially designed to assess symptom severity according to DSM-IV criteria. Items are rated on a 4-point Likert scale ranging from 0 (not at all) to 3 (nearly every day), and total scores range from 0 to 21. The total score is interpreted as “minimal anxiety” if 0–4, as “mild anxiety” if 5–9, as “moderate anxiety” if 10–14, and as “severe anxiety” if 15–21, according to the original authors. The cut-off value for identifying cases of GAD is at 10 points.

##### 2.3.2.2 Beck depression inventory

To assess the presence and severity of depressive symptoms, the Beck Depression Inventory (2nd Edition) was used. This self-report scale comprises 21 items. Items are rated on a 4-point Likert scale ranging from 0 to 3, reflecting increasing severity of depression. Total scores range from 0 (“Never”) to 3 (“Nearly every day”), and total scores range from 0 to 63. The total scores of 0 to 9 indicate “minimal depression”, scores of 10 to 18 suggest “mild depression”, scores of 19 to 29 suggest “moderate depression”, and scores of 30 to 63 suggest “severe depression”.

##### 2.3.2.3 General health questionnaire

The GHQ-12 by Goldberg (21) records short-term changes in the state of health. There is a German version of the GHQ-12 (26) and a validated, standardized Russian version (25). This was also used in Ukraine, since all participants spoke Russian. Symptoms such as anxiety, sleep disturbances, or physical discomfort from the last 14 days are registered in this questionnaire. The self-administered questionnaire collects a total of 12 items using a 4-point Likert scoring method, with the range of 0 =“less than usual”, 1= “no more than usual”, 2= “rather more than usual”, 3 = “much more than usual”. Positive questions (items 1, 3, 4, 7, and 12) were scored inversely. Participants in the intervention group completed a baseline assessment approximately one week before the logotherapy sessions began. A post-intervention assessment was administered within one week after the final session, resulting in a total participation period of approximately seven to eight weeks. The control group followed the same assessment schedule (baseline and follow-up six weeks apart) without receiving any treatment during that time.

### 2.4 Statistical analysis

Results were analyzed using GraphPad by Dotmatics. Data distributions were visually inspected using Q-Q plots and histograms to assess normality; no major deviations were observed. Differences between the two independent samples were analyzed using the Student *t*-test, whereas the paired *t*-test was used to analyze within-group differences over time. Chi-square and Fisher’s exact test were used to analyze frequencies. We also used effect sizes (Cohen’s d) to contextualize the magnitude of the changes. Statistical significance was considered at p < 0.05 in all cases.

### 2.5 Ethical considerations

This study was conducted in accordance with the ethical standards of the Declaration of Helsinki and the Barcelona. All participants received a printed explanatory statement which included information about the study’s purpose, voluntary participation, potential risks, and confidentiality. Informed consent was obtained prior to participation. Confidentiality was protected in online group sessions, with no recordings, all responses were anonymized, and no personally identifying information was collected beyond general demographic characteristics. Data were stored on a password-protected, secure cloud server accessible only to the research team. A distress protocol was available during group sessions, and risk was monitored during and after the sessions. Participants could access support after the intervention by contacting the research team/facilitator.

## 3 Results

### 3.1 Design of the logotherapy intervention protocol

**Figure 1:**
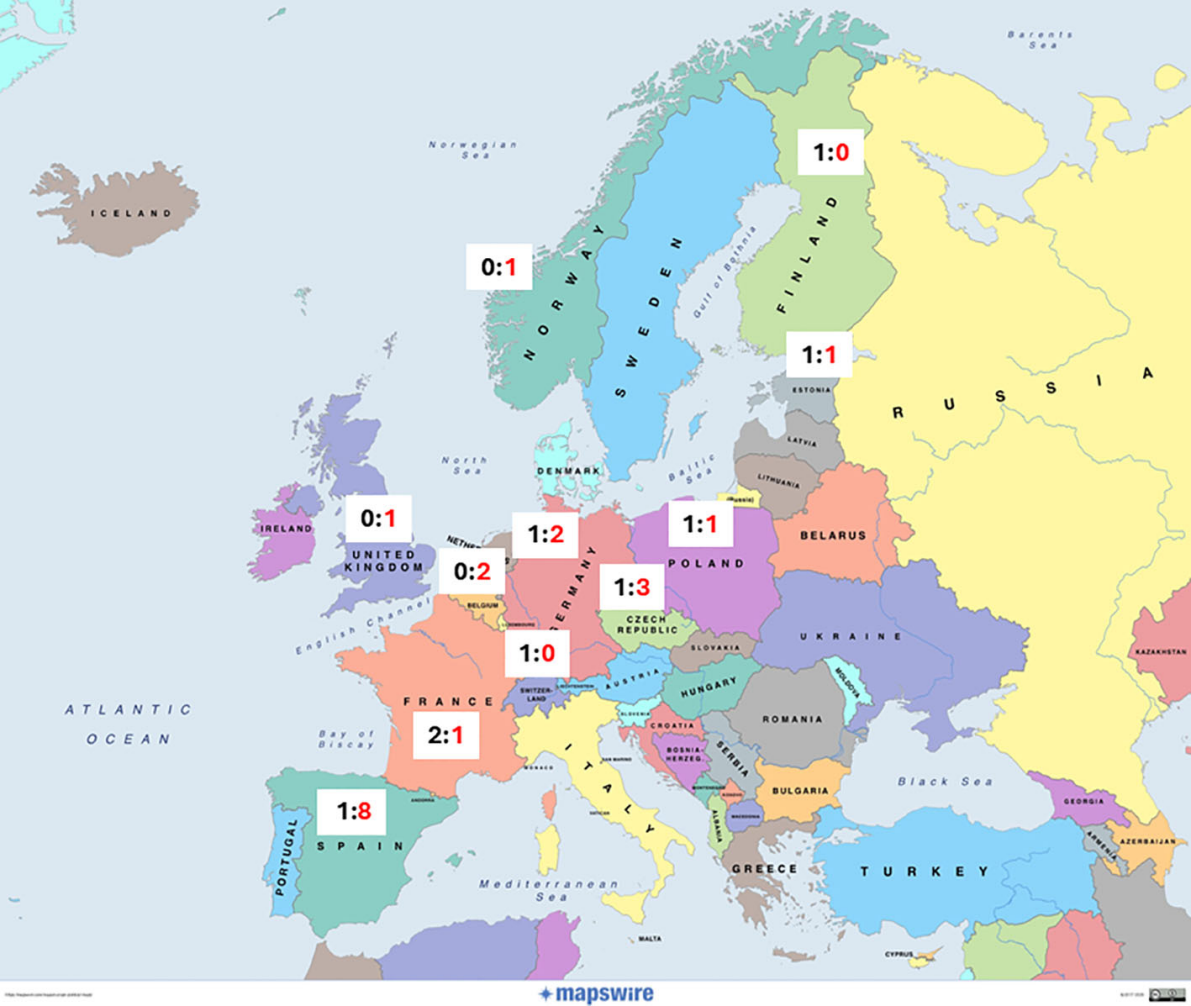
Geographical distribution in Europe of Ukrainian refugee participants Distribution depicted in a European political map, Miller by apswire.com CC-BY 4.0. In each country with participants, the box indicates the ratio (black: red) of participants in the control group (black) versus the logotherapy group (red).

Based on our prior study of the young Iranian population in Europe (11), a short-term protocol was designed comprising six closed-group sessions, each lasting approximately 90 minutes, with topics and tools as described below (**Table 1**). Sessions were conducted either online or in person, depending on participants’ accessibility and location, as their geographical distribution spanned 8–9 European countries (**Figure 1**).

**Table 1:** A structured overview of the six-session logotherapy protocol for Ukrainian **r**efugees.

Logotherapy intervention protocol for Ukrainian refugees				
Session	Objective	Key activities	Assignments	Techniques
1 Introduction to Meaning of Life and Orientation	Establish therapeutic alliance; Introduce logotherapy and the concept of meaning. Explore current challenges related to displacement using examples from Ukrainian history and literature.	Psychoeducation on meaning, Freedom of attitude. guided discussion about personal experiences of migration and loss. Initial Socratic exploration of inner resources.	Reflective journaling: “What gives me a sense of meaning now?”	Socratic Dialogue, Cognitive Meaning-Orientation, Attitudinal Adjustment
2 Exploring Existential Concerns, Values Clarification and Personal Strengths	Identify core values and personal strengths that persist despite displacement.	Values-identification exercises. Discussion of past moments when the participant experienced meaning. Exploration of strengths and resilience factors.	A short written list of three personal values and examples of how they manifest.	Socratic Dialogue, Dereflection (shifting focus to strengths), Meaning Exploration
3 Introspection Attitudinal Work and Reframing of Suffering	Support participants in re-evaluating unavoidable suffering through meaning-oriented attitudes.	Guided reflection on uncontrollable vs. controllable aspects of their current life. Narrative exercises about overcoming difficulties. Group or individual discussion.	“Reframing task”: Describe one difficult experience and identify a meaningful attitude toward it.	Attitudinal Modification, Socratic Dialogue
4 Self-Awareness and Growth Overcoming barriers and inner blocks	Address emotional or cognitive blocks that inhibit meaning-making.	Sharing of life experiences was framed within Ukrainian collective cultural values and dysfunctional worry patterns. Exploration of anxiety triggers. Exercises to redirect attention toward purposeful action.	Practice noticing “meaningful moments” during the week.	Dereflection, Cognitive Meaning-Orientation
5 Empowerment and Facing Challenges Future Meaning and Goal Reconstruction	Strengthen a sense of agency and future purpose. Build medium-term goals.	A “time travel” exercise incorporated Ukrainian culturally meaningful images of resilience and national identity. Discussion of realistic, purposeful steps. Mapping personal aspirations despite current uncertainty.	Set one concrete action aligned with a personal value.	Meaning Reconstruction, Socratic Dialogue
6 Meaning of Life and Therapy Conclusion	Summarize progress. Integrate learning. Reinforce commitment to meaning-centred living.	Review of changes across six weeks, used culturally significant Ukrainian metaphors and symbols to help participants articulate personal meaning and synthesize therapeutic insights. Discussion of maintaining meaningful attitudes after the intervention.	Optional: Write a short “meaning statement” summarizing insights.	Attitudinal Adjustment, Socratic Dialogue, Dereflection

#### 3.1.1 Session structure and objectives

The sessions were structured to foster engagement and meaningful participation. They aimed to progressively introduce and build upon foundational concepts, allowing participants to reflect deeply on personal experiences and existential concerns. Key logotherapy techniques—including Socratic Dialogue, Modification of Attitude, Paradoxical Intention, and Dereflection—were integrated throughout each session to address various aspects of mental well-being. The control group did not attend the sessions, and no specific psychological treatment was administered to this group.

#### 3.1.2 Cultural adaptation

To enhance cultural relevance and engagement, the intervention was tailored to the lived experiences of Ukrainian refugees. Session content included culturally resonant examples, metaphors, and existential themes related to war, displacement, and national identity. Discussions were conducted in Ukrainian or Russian, depending on participants’ preference, to ensure emotional comfort and comprehension. Exercises encouraged reflection on personal values, loss, and resilience within a culturally familiar framework. The facilitator integrated Ukrainian literary references and culturally significant symbols to support participants’ connection to meaning and purpose.

#### 3.1.3 Facilitator training

The facilitator was a psychologist with clinical training and prior experience working with trauma-affected populations and a native speaker of Ukrainian and Russian. The facilitator was also briefed on ethical considerations and cultural sensitivities related to working with war-affected populations.

#### 3.1.4 Fidelity monitoring

Although this pilot study did not include formal audio/video recordings or third-party fidelity assessments due to logistical constraints, fidelity was supported through the following mechanisms:

- A detailed facilitator manual with structured session outlines and key objectives.
- Session checklists completed by facilitators after each group meeting to record adherence to the planned content and techniques (e.g., Socratic Dialogue, Paradoxical Intention, Modification of attitude, Dereflection).
- Post-session written reflections to capture challenges, participant engagement, and perceived impact.

### 3.2 Sample of participants

After recruitment, a total of 23 individuals were initially assigned to the logotherapy intervention group; 20 completed the full protocol, and 3 participants dropped out. The control group consisted of nine individuals recruited separately from the same general refugee population, but they did not receive any psychological intervention. The study was performed from December/2022 to May/2023 with a wide geographical distribution involving 11 European countries (Spain, France, Belgium, Switzerland, Czechoslovakia, UK, Germany, Poland, Estonia, Norway, Finland). Participants suffered from moderate anxiety, mild depression, and possible recent appearance of mental health problems according to their pre-tests.

### 3.3 Sociodemographic indicators of the logotherapy and control groups

From the total of 23 individuals that were initially assigned to the logotherapy intervention group, 20 completed the full protocol and three participants dropped out. The control group consisted of nine individuals recruited separately from the same general refugee population, but they did not receive any psychological intervention.

**Table 2** presents the sociodemographic characteristics of the sample. The majority of participants were women (only 1 man in each group), and their average ages were comparable across groups, despite wide age ranges (27–46 in the control group; 18–62 in the logotherapy group). Marital status, level of education, presence of chronic diseases, place of residence before the war, and with whom relatives left Ukraine - all these indicators were comparable between the two groups. Still, in the control group, all participants had higher education and were from large cities, whereas in the logotherapy group, some had vocational or secondary education and were from smaller cities. Statistically significant quantitative differences were found in the duration of respondents’ stay abroad after the beginning of the war in Ukraine, with longer durations in the control group (10.8 ± 0.4 months) than in the logotherapy group (8.4 ± 0.3 months; p = 0.0001).

**Table 2:** Social demographic indicators of the logotherapy and control groups.

	Descriptives	Control group (n=9)	Logotherapy group (n= 20)	Statistics student *t-*test chi-square (Χ^2^)
Sociodemographic variables				
Sex/Gender	Women: Men	8:1	19:1	
Age	Years old (mean ± SEM) Range	34.4 ± 2.4 27-46	38.9 ± 2.5 18-62	Student *t-*test *n.s.*
Marital status	Single	5	4	Χ^2^ = 5.27, 3 df p = 0.15
	Divorced	0	5	
	Married	4	10	
	Widower	0	1	
Education	General education	0	1	Χ^2^ = 2.09, 2 df p = 0.35
	Secondary special education	0	3	
	Higher education	9	16	
Have a chronic disease	Yes	5	12	Χ^2^ = 0.05, 1df p = 0.82
	No	4	8	
Place of residence in Ukraine before war	A small city (< 50.000 people)	0	2	Χ^2^ = 4.97, 3 df p = 0.17
	A medium-sized city (50.000 – 250.000 people)	0	2	
	A medium-sized city (250.000 – 500.000 people)	0	4	
	A large-sized city (> 500.000 people)	9	12	
Refugee status				
Did you lose a close person in wartime	Yes	6	2	Χ^2^ = 9.98, 1 df p = 0.002**
	No	3	18	
You went abroad with.	parents	1	2	Χ^2^ = 3.30, 5 df p = 0.65
	children	2	6	
	children and/or husband	2	4	
	other relatives	0	4	
	friends	1	1	
	Came alone	3	3	
Time abroad	Months (Mean + - SEM)	10.8 ± 0.4	8.4 ± 0.3	Student *t-*test p < 0.001***
Country destination	Geographic representation (total number of countries)	8	9	(see **Figure 1**)

Data is expressed as ratio, mean ± SEM, range, or incidence. Control group, n = 9; Logotherapy group, n = 20. Between-group comparison, Student *t-*test, **p < 0.01, ***p < 0.001 logotherapy vs. controls.

The profile of the three participants that dropped out was heterogeneous for marital status (1 married, 2 divorced), education (1 secondary education, 2 higher education), existence of a chronic disease (2 with chronic disease, 1 without), with whom they went abroad (2 with children, 1 with other relative) and the only coincidence was that they had no lost any person in wartime, and were from a large city. With regards to clinical assessments during the interview, they exhibited mild anxiety (GAD-7 score 5.00 ± 2.65) and mild depressive symptoms (BDI-II score 18.00 ± 9.87), while their GHQ-12 score (5.33 ± 1.76) suggested a general normal mental health status.

### 3.4 Logotherapy intervention protocol in Ukrainian refugees

While individual item-level changes are presented only for descriptive purposes, the primary interpretation focuses on total score changes on validated scales (GAD-7, BDI, and GHQ-12). Given the exploratory nature of this pilot study and the relatively small sample size, no correction for multiple comparisons was applied, and p-values for individual items should be interpreted with caution.

#### 3.4.1 Reduction of anxiety levels as assessed by GAD-7

The total anxiety score at baseline (**Table 3A**) did not differ between groups (p = 0.2575), and none of the items differed between groups either (all p > 0.05).

**Table 3:** Specific and total GAD-7, BDI and GHQ-12 scoring distribution in the control and logotherapy groups.

	Control group		Logotherapy group		
A. GAD-7 During the last two weeks, were you …	GAD-7 Pre	GAD-7 Post	GAD-7 Pre	GAD-7 Post
… feeling nervous, anxious, or on edge?	1.00 ± 0.33	1.33 ± 0.37	1.70 ± 0.23	1.25 ± 0.16 *
… not being able to stop or control worrying?	1.56 ± 0.18	0.78 ± 0.15 *	1.65 ± 0.22	0.95 ± 0.05 **
… had trouble relaxing?	0.89 ± 0.26	0.67 ± 0.24	1.25 ± 0.23	0.75 ± 0.18 *
… worrying too much about different things?	1.11 ± 0.35	1.33 ± 0.29	1.55 ± 0.25	1.10 ± 0.18
… being so restless that it’s hard to sit still?	0.44 ± 0.24	0.67 ± 0.24	0.80 ± 0.21	0.40 ± 0.15
… becoming easily annoyed or irritable?	1.00 ± 0.33	0.67 ± 0.24	1.35 ± 0.21	0.55 ± 0.17 **
… feeling afraid as if something awful might happen?	0.78 ± 0.27	0.33 ± 0.17 *	1.15 ± 0.24	0.75 ± 0.22
Total score GAD-7	6.78 ± 1.79	5.78 ± 1.12	9.45 ± 1.32	5.75 ± 0.74 ***
B. BDI questions	BDI Pre	BDI Post	BDI Pre	BDI Post
1. Sadness	0.44 ± 0.34	0.56 ± 0.18	1.00 ± 0.21	0.40 ± 0.17 *
2. Pessimism	0.44 ± 0.18	0.22 ± 0.15	0.75 ± 0.20	0.40 ± 0.11 *
3. Past Failure	0.22 ± 0.15	0.22 ± 0.15	0.45 ± 0.18	0.05 ± 0.05
4. Loss of Pleasure	0.44 ± 0.18	0.44 ± 0.18	1.20 ± 0.19 c	0.80 ± 0.16
5. Guilty Feelings	0.33 ± 0.17	0.67 ± 0.33	0.95 ± 0.18 c	0.30 ± 0.11 **
6. Punishment Feelings	0.44 ± 0.18	0.56 ± 0.24	0.95 ± 0.17	0.60 ± 0.15 *
7. Self-Dislike	0.00 ± 0.00	0.22 ± 0.15	0.25 ± 0.16	0.15 ± 0.15
8. Self-Criticalness	0.67 ± 0.17	0.44 ± 0.24	0.65 ± 0.17	0.25 ± 0.10 *
9. Suicidal Thoughts or Wishes	0.33 ± 0.17	0.33 ± 0.17	0.60 ± 0.15	0.25 ± 0.10 *
10. Crying	0.22 ± 0.15	0.44 ± 0.18	0.20 ± 0.09	0.05 ± 0.05 c
11. Agitation	1.11 ± 0.26	0.89 ± 0.11	1.05 ± 0.17	0.85 ± 0.08
12. Loss of Interest	0.56 ± 0.18	0.67 ± 0.24	1.00 ± 0.22	0.45 ± 0.17 *
13. Indecisiveness	0.89 ± 0.20	0.78 ± 0.15	1.25 ± 0.16	0.95 ± 0.18
14. Worthlessness	0.56 ± 0.24	0.33 ± 0.17	0.65 ± 0.17	0.30 ± 0.13
15. Loss of Energy	0.78 ± 0.22	0.78 ± 0.22	1.00 ± 0.22	0.55 ± 0.15 *
16. Changes in Sleeping Pattern	1.33 ± 0.24	1.00 ± 0.29	1.45 ± 0.25	0.60 ± 0.18 **
17. Irritability	0.78 ± 0.22	1.00 ± 0.24	0.95 ± 0.22	0.10 ± 0.07 **c
18. Changes in Appetite	0.33 ± 0.24	0.33 ± 0.17	0.35 ± 0.17	0.25 ± 0.16
19. Concentration Difficulty	0.56 ± 0.24	0.78 ± 0.28	0.50 ± 0.14	0.15 ± 0.08 *c
20. Tiredness or Fatigue	0.89 ± 0.26	0.56 ± 0.24	1.00 ± 0.24	0.80 ± 0.21
21. Loss of Interest in Sex	0.78 ± 0.22	0.33 ± 0.17	0.85 ± 0.22	0.65 ± 0.20
Total BDI score	12.11 ± 1.97	11.56 ± 2.64	17.05 ± 2.56	3.90 ± 1.55 **
C. GHQ-12 Have you recently…	GHQ-12 Pre	GHQ-12 Post	GHQ-12 Pre	GHQ-12 Post
… been able to concentrate on what you’re doing?	0.22 ± 0.15	0.22 ± 0.15	0.35 ± 0.12	0.20 ± 0.12
… lost much sleep over worry?	0.44 ± 0.24	0.67 ± 0.24	0.52 ± 0.15	0.85 ± 0.21
… felt that you’re playing a useful part in things?	0.00 ± 0.00	0.22 ± 0.15	0.17 ± 0.10 c	0.15 ± 0.08
… felt capable of making decisions about things?	0.00 ± 0.00	0.00 ± 0.00	0.04 ± 0.04	0.05 ± 0.05
… felt that you’re enjoying your normal day-to-day activities?	1.11 ± 0.11	1.44 ± 0.18	0.83 ± 0.16	0.85 ± 0.17 c
… been losing confidence in yourself?	0.22 ± 0.15	0.44 ± 0.18	0.61 ± 0.15 c	0.30 ± 0.11 *
… felt able to face your problems?	0.33 ± 0.24	0.11 ± 0.11	0.35 ± 0.10	0.25 ± 0.10 *
… been feeling unhappy or depressed?	0.89 ± 0.31	0.78 ± 0.22	1.17 ± 0.21	0.75 ± 0.16
… felt you could not overcome your difficulties?	1.33 ± 0.24	1.44 ± 0.29	1.43 ± 0.22	1.30 ± 0.18
… been able to face up to your problems?	1.00 ± 0.00	1.11 ± 0.11	1.52 ± 0.15 c	1.25 ± 0.12
… been thinking of yourself as a worthless person?	0.00 ± 0.00	0.11 ± 0.11	0.13 ± 0.10	0.05 ± 0.05
… felt reasonably happy, all things considered?	0.78 ± 0.22	0.56 ± 0.18	0.65 ± 0.16	0.55 ± 0.14
Total GHQ-12 score	6.33 ± 0.44	7.11 ± 0.61	7.80 ± 0.85	6.55 ± 0.58

Data is expressed as mean +_ SEM. Control group, n = 9; Logotherapy group, n = 20. Exploratory analysis: Between-group comparison, Student *t*-test, cp < 0.05, logotherapy vs. controls. Within groups, Pre-post paired *t*-test, statistical values *p < 0.05, **p < 0.01, ***p < 0.001 vs. baseline in their first survey. Pre-post paired *t-*test in the control group with p = 1.000.

In evaluating the pre-post in the control group, we did not observe any significant changes in the total score (p = 0.4021), nor in most of the indicators of anxiety as assessed by the GAD-7 questionnaire (p > 0.05) except for the ability to control and stop anxious feelings (decrease, p = 0.02) and feeling afraid as if something awful might happen (decrease, p = 0.04). In contrast, the participants in the logotherapy intervention experienced a statistically significant decrease in the total anxiety score levels (p = 0.0005), particularly due to the improvements of their answers to questions describing (in order of statistical significance) a higher ability to stop or control anxious feelings (p = 0.0093), a lower threshold of irritability (p = 0.0043), less problems with relaxation (p = 0.0210) or feeling anxious (p = 0.0248) (**Table 3A**).

#### 3.4.2 Reduction of depression levels as assessed by BDI

The total depression score at baseline (**Table 3B**) did not differ between groups (p = 0.2348). However, there were statistically significant differences in responses to questions such as ‘Loss of pleasure’ and ‘Guilty feelings’, with higher basal values in the logotherapy group than in controls (p < 0.05).

In evaluating the pre-post in the control group, we did not observe any significant changes in the total score. In the item analysis, some questions (No.3, 4, 9, 15, 18) did not change at all (p = 1.000), and most of the others suggested an unfavourable dynamic in respondents’ mental state between the first (pre) and final (post) surveys.

Among participants in the logotherapy intervention, the pre-post analysis showed a statistically significant decrease of 9 points in the total depression score (p = 0.0097) and improvement in half of the symptoms (11 of 21) (**Table 3B**). They experienced (in order of statistical significance) fewer feelings of guilt, reduced changes in sleep pattern and irritability (p < 0.01); they reduced suicidal thought, they were less concerned about feelings of sadness and anxiety regarding their future, reduced punishment feelings, decreased self-criticalness and the loss of interest and energy, their difficulties to concentrate improving their ability to be productive at work (p < 0.05).

At the end of the logotherapy intervention, participants exhibited a statistically significant reduction of crying, irritability, and difficulty concentrating as compared to the control. It is also noteworthy that while suicidal thoughts were sustained over time in the control group (p = 1.000), the participants in the logotherapy group experienced a significant im4rovement in this aspect.

#### 3.4.3 Improvement of general health as assessed by GHQ-12

At baseline, the total general health score (**Table 3C**) did not differ significantly between the groups (p = 0.2724), and the values were within the range indicating a possible presence of mental health difficulties. Most GHQ-12 items were scored in the range of 0–2 (0 = “less than usual”, 1 = “no more than usual”, 2 = “rather more than usual”). Overall, participants in both groups showed similar response patterns across most items. However, three items differed significantly between groups. Participants in the logotherapy group reported a stronger perception of having a useful role and a greater ability to face their problems than controls (p < 0.05). At the same time, they reported more frequently having recently lost confidence in themselves (p < 0.05).

The generally moderate responses across many items, despite the stressful context of forced migration, may reflect difficulties in emotional awareness or expression. One possible interpretation is the presence of alexithymic tendencies, where individuals experience distress but have a limited ability to identify or verbalize their emotional states. In the context of displacement, this pattern may arise when individuals feel psychologically overwhelmed as they attempt to adapt to major life changes, including altered lifestyles, disrupted social roles, and the need to secure basic resources for survival.

In the control group it is interesting to note that the respondents answered negatively (0=‘less than usual’) to 3 of the 12 questions: “Have you recently … felt that you’re playing a useful part in things? … capable of making decisions about things? … been thinking of yourself as a worthless person?” resulting in a zero score for these indicators. The pre-post observation comparison showed that ‘decision making’ remained at a null value, while for the other two items, some respondents scored the questions with 1 = “no more than usual’.

After the logotherapy intervention, statistically significant differences were observed only in two items: one assessing self-confidence (less loss of self-confidence, p < 0.05) and the other assessing the ability to face problems (reduced, p < 0.05). Overall, about the other half of the items (5 out of 12) showed a trend towards worsening, while the other half (5 out of 12) showed improvement.

### 3.5 Comparative GAD-7, BDI and GHQ-12 scoring distribution

**Figure 2:**
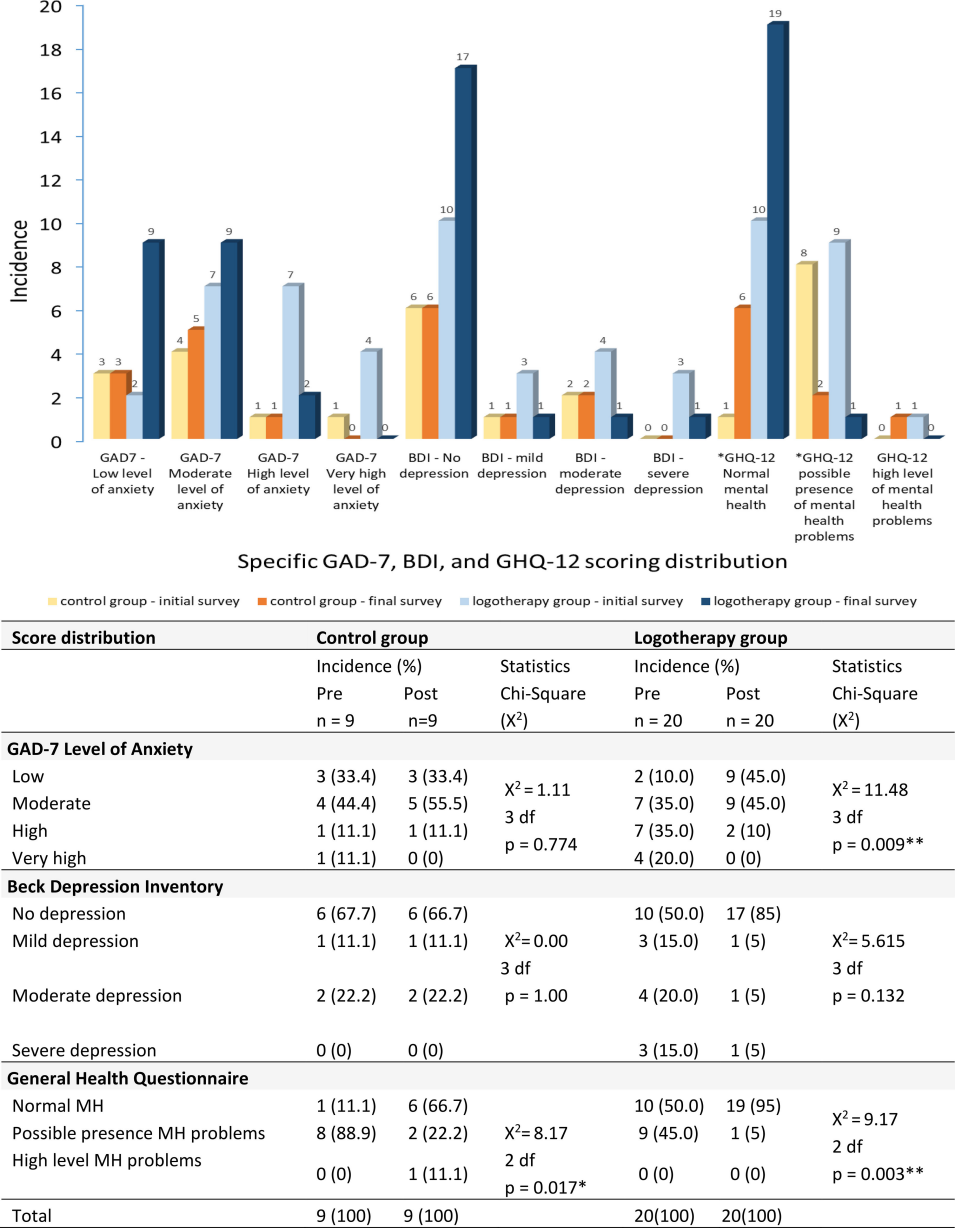
Comparison of specific GAD-7, BDI, and GHQ-12 scoring distribution. Data is expressed as incidence per GAD-7, BDI and GHQ-12 score distribution in the control (orange bars, n=9) and logotherapy (blue bars, n=20) groups at the initial (light color bars) and final (dark color bars) survey. Exploratory statistical analysis is depicted in the table, were data is expressed as incidence and percentage. Control group, n = 9; Logotherapy group, n = 20. Within-group pre-post comparison, Chi-square, red map, *p < 0.05, **p < 0.01 vs initial survey.

A significant improvement was observed across all assessments following the logotherapy sessions (**Figure 2**, dark blue bars). The number of participants presenting severe levels of psychological disorders decreased notably, while the proportion of individuals with no symptoms or only minimal manifestations increased. In contrast, the control group exhibited some minor changes in mental health status, that did not reach statistical significance.

More detailed information on changes in the distribution’s score is presented in **Figure 2**. Statistically significant improvements were observed in GAD-7 scores, indicating a reduction in anxiety levels following the logotherapy sessions and reflecting enhanced mental health among participants. For the BDI and GHQ assessments, significant changes were found only in selected indicators following the intervention.

To quantify the magnitude of the observed changes, Cohen’s d with 95% confidence intervals was calculated for pre–post comparisons. In the Logotherapy group, effect sizes indicated a medium reduction in anxiety symptoms measured by GAD-7 [d = 0.64, 95% CI (0.02, 1.25)], a large reduction in depressive symptoms on the Beck Depression Inventory [d = 0.81, 95% CI (0.18, 1.43)], and a small reduction in general distress measured by GHQ-12 [d = 0.38, 95% CI (−0.22, 0.99)]. In the control group (no psychotherapy, baseline vs. 6 weeks), effect sizes were small and not statistically precise: GAD-7 [d = 0.22, 95% CI (−0.70, 1.15)], BDI [d = 0.08, 95% CI (−0.84, 1.00)], and GHQ-12 [d = −0.49, 95% CI (−1.43, 0.45)]. Effect sizes indicated medium reductions in anxiety and large reductions in depressive symptoms, although confidence intervals were wide due to sample size.

## 4 Discussion

The American Psychiatric Association (14) has emphasized the importance of carefully considering the manifestation and severity of psychiatric/psychological symptoms in refugee communities, regardless of whether they are related to an existing disorder or a prodromal phase. We implemented this idea in our study and would like to compare our results with previous studies.

The literature on providing psychological assistance to refugees mostly focuses on post-traumatic stress disorder and trauma-related symptoms, with less attention given to depression and anxiety disorders. Thus, general cognitive-behavioral therapy, narrative exposure therapy, and a variety of integrative and interpersonal therapies have been used to provide assistance (27). However, techniques of logotherapy can help clients achieve long-term relief from major stressors associated with refugees and settling in a new place (10). They are also effective for individuals from diverse cultures and religions (11). In the present quasi-experimental work, our logotherapy protocol for Iranian international students, shown to be effective in improving their anxiety and depressive symptoms during the COVID-19 Pandemic (10), was adapted and tailored to the community setting of Ukrainian refugees established in Barcelona and extended to those welcomed to other European destinations. We focused specifically on anxiety and depressive symptoms, which are prevalent and universal symptoms of many mental disorders. Other authors (28) have also highlighted the effectiveness and potential of logotherapy among Ukrainian refugees. In their study it was shown that although in this population a clear diagnosis of depression according to the Diagnostic and Statistical Manual of Mental Disorders - Fifth Edition (DSM-V) was often difficult to establish, what did emerge prominently were the themes representing the five sub-components of demoralization, namely loss of meaning of life, hopelessness or disheartenment, helplessness, sense of failure, and dysphoria. Hence, given that the loss of meaning/purpose in life is a crucial subcomponent of demoralization, conducting meaning-oriented therapy could be an intervention in community settings.

By applying key logotherapy techniques among refugees from Ukraine, we have observed positive changes in the respondents’ conditions in anxiety and depressive symptoms. In this respect, the GHQ-12 was less sensitive to changes, probably because the questionnaire is done in terms of comparison with the prior situation, or norm. Considering that refugees were exposed to severe scenarios, null values (‘less than usual’) may reflect relief from the war, but could also suggest alexithymia. Although our work was pilot in nature and involved a small number of respondents, a number of statistically significant results in anxiety and depression symptoms suggest that logotherapy techniques deserve greater attention in organizing psychological assistance for refugees.

The gender composition of our study was predominantly female, which is not surprising since refugees are mostly women, children, and older individuals who cannot engage in combat. Men, on the other hand, are more likely to participate in combat, as confirmed by earlier publications (29). Although women were also recruited as militiamen in the current war, mobilization primarily involved the country’s male population, as during the waves of mobilization at the beginning of the conflict in 2014 (1).

The average age of participants was comparable between groups and centered in their third decade. Although group assignment was not randomized, these values may reflect the broader age distribution among Ukrainian refugees displaced by the current military conflict. Given the wide age range of 18 to 65 years, it is important to consider that age could moderate the effectiveness of meaning-centered interventions. Older individuals may draw more extensively on retrospective reflection and life experience, whereas younger participants might focus more on future-oriented meaning-making. While the present study was not powered to test age as a moderating variable, future research should systematically investigate this relationship. A deeper understanding of age-related developmental factors could inform the tailoring of logotherapy interventions to enhance their efficacy across different age groups.

The present pilot study results on depression and anxiety symptoms among Ukrainian refugees across multiple standardized self-report measures, align with existing literature highlighting the elevated risk of mental health disorders in refugee populations, including anxiety, depression, and PTSD. In fact, the systematic review and meta-analysis of WHO prevalence estimates of mental disorders in conflict settings (30), reported that approximately one in five individuals affected by armed conflict suffer from a mental disorder, with 5.1% experiencing severe forms and 17% experiencing mild to moderate disorders. In our study, high and very high levels of anxiety were reported by 26% and 22% of participants in the logotherapy group prior to intervention, compared to 11.5% in the control group. Additionally, severe depressive symptoms were observed in 13% of the logotherapy group. While self-assessment instruments may overestimate the prevalence of disorders—potentially inflating rates by 1.5 to 2 times due to their lack of diagnostic specificity—these tools nonetheless reflect participants’ perceived mental health burden. Notably, following the logotherapy intervention, the exploratory analysis showed statistically significant reductions were observed in both anxiety and depressive symptom scores, underscoring the potential utility of logotherapy in alleviating psychological distress in trauma-affected populations.

During times of war, there are two categories of refugees: internally displaced persons and refugees in other countries. A study of internally displaced persons revealed a high prevalence of mental disorders in this category of refugees, which is consistent with the results we obtained. According to the findings of a study by Roberts et al. (31), only 26% of refugees suffering from mental disorders sought medical help for their condition. The results of our study suggest a positive trend in the context of group psychotherapy, indicating its potential as a model for providing psychological support to refugee populations.

Comprehensive MHPSS (Mental Health and Psychosocial Support) programs for refugees combine somatic health issues, social support, education, and targeted psychiatric/psychological interventions to address the problem of increasing mental health disorders. The engagement of Ukrainian-speaking specialists, as implemented in the current pilot project on the potential of logotherapy, is considered highly important for the effective use of resources in assisting refugees (32).

This pilot study was exploratory in nature, aiming to assess the feasibility and preliminary impact of logotherapy among Ukrainian refugees under real-world constraints. This study has several limitations related to small sample size, group imbalance and confounding factors that must be acknowledged. First, the final sample comprised 23 participants in the intervention group (of whom 20 completed the post-intervention assessments) and 9 in the control group. This affects statistical power and limits the generalizability of the findings. Despite this, the exploratory analysis pointed to several statistically significant findings that provide early evidence of potential therapeutic benefits and support the need for larger-scale, randomized studies. The second limitation was that the group assignment was non-randomized, and no baseline matching for some of their sociodemographic variables was performed, resulting in statistically significant differences in educational background and urban/rural origin between groups. The sample was too small to adjust the analyses for key baseline differences in educational and sociodemographic determinants, so causal interpretation in this respect is not possible. Since the groups were not equivalent, baseline imbalances in some variables may have introduced confounding and influenced symptom trajectories. Other unmeasured confounders would include social support, employment, housing stability, host-country conditions, trauma exposure, current stressors, access to services, and prior mental health history. However, it is also important to note that the total score for anxiety, depression, and general health at baseline did not show differences between the logotherapy and control groups. Finally, the third limitation concerns the methods and tools. The present work reports fidelity checklists and facilitator reflections, but no independent fidelity assessment. This should be clearly described as a limitation. With regards to the tools, the three psychodiagnostics methods (GAD-7, BDI and GHQ-12) are recognized as valid and reliable in the healthcare system of Ukraine in accordance with the Order of the Ministry of Health of Ukraine dated December 13, 2023, No. 2118 (23), which defines the list of valid methods of psychological diagnostics that can be used to conduct psychological diagnostics and assess the quality of psychological care. However, despite the Russian version of the GHQ-12 being used to accommodate participants’ fluency, we recognize that political and cultural sensitivities related to the ongoing conflict may have influenced emotional engagement or response bias. We also recognize the importance of ongoing supervision and fidelity monitoring in psychological intervention research. Future studies will include systematic fidelity checks, session recordings (with consent), and regular supervision to ensure consistency and quality of implementation. Finally, several of the cited references were drawn from Russian-language publications that may lack transparent peer-review processes; we have taken steps to clarify their origins and, where possible, support them with internationally recognized sources. Future research should address these methodological constraints using larger, randomized, and culturally sensitive study designs.

## 5 Conclusions

This pilot study suggests the feasibility, acceptability, and cultural adaptation of logotherapy for Ukrainian refugees. Despite challenging conditions, the intervention was delivered successfully with high completion and engagement, supporting its practicality in real-world humanitarian settings. The exploratory analysis indicated that medium to large within-group improvements were observed for anxiety and depression, though general distress changes were smaller and less consistent. These findings should be interpreted as preliminary, given the wide confidence intervals, small sample size, and non-randomized design. In addition, multiple item-level comparisons without adjustment increase the risk of Type I error. Such limitations are common in early-phase exploratory research but underscore the need for larger, randomized, and methodologically rigorous trials. Future studies should apply corrections for multiple testing, consider non-parametric approaches when statistical assumptions are not met, and incorporate systematic fidelity monitoring to more robustly evaluate efficacy. Finally, the study measures outcomes only immediately after the six-week intervention. Therefore, the durability of effects is unknown, and future studies require medium- and longer-term follow-up.

## Data Availability

The original contributions presented in the study are included in the article/supplementary material. Further inquiries can be directed to the corresponding author.
